# Supplementary material for: Inhibition and transport mechanisms of the ABC transporter hMRP5
Source: Nat Commun. 2024 Jun 6;15:4811. doi: 10.1038/s41467-024-49204-1 (PMC11156954; doi:10.1038/s41467-024-49204-1)

Supplementary Information

Inhibition and transport mechanisms of the ABC transporter hMRP5

Ying Huang[1,4#], Chenyang Xue[1,4#], Ruiqian Bu[1,4#], Cang Wu[1,4#], Jiachen Li[2], Jinqiu Zhang[2], Jinyu Chen[2], Zhaoying Shi[6], Yonglong Chen[6], Yong Wang[2,3*], Zhongmin Liu[1,4*]

[1]Shenzhen Key Labortory of Biomolecular Assembling and Regulation, School of Life Sciences, Southern University of Science and Technology, Shenzhen 518055, Guangdong, China

[2]College of Life Sciences, Zhejiang University, Hangzhou 310027, China

[3]The Provincial International Science and Technology Cooperation Base on Engineering Biology, International Campus of Zhejiang University, Haining, 314400, China

[4]Department of Immunology and Microbiology, School of Life Sciences, Southern University of Science and Technology, Shenzhen 518055, Guangdong, China

[5]Institute for Biological Electron Microscopy, Southern University of Science and Technology, Shenzhen 518055, Guangdong, China

[6]Department Of Chemical Biology, School of Life Sciences, Southern University of Science and Technology, Shenzhen 518055, Guangdong, China

[#]These authors contributed equally to this work

*Correspondence and lead contact: yongwang_isb@zju.edu.cn, liuzm@sustech.edu.cn

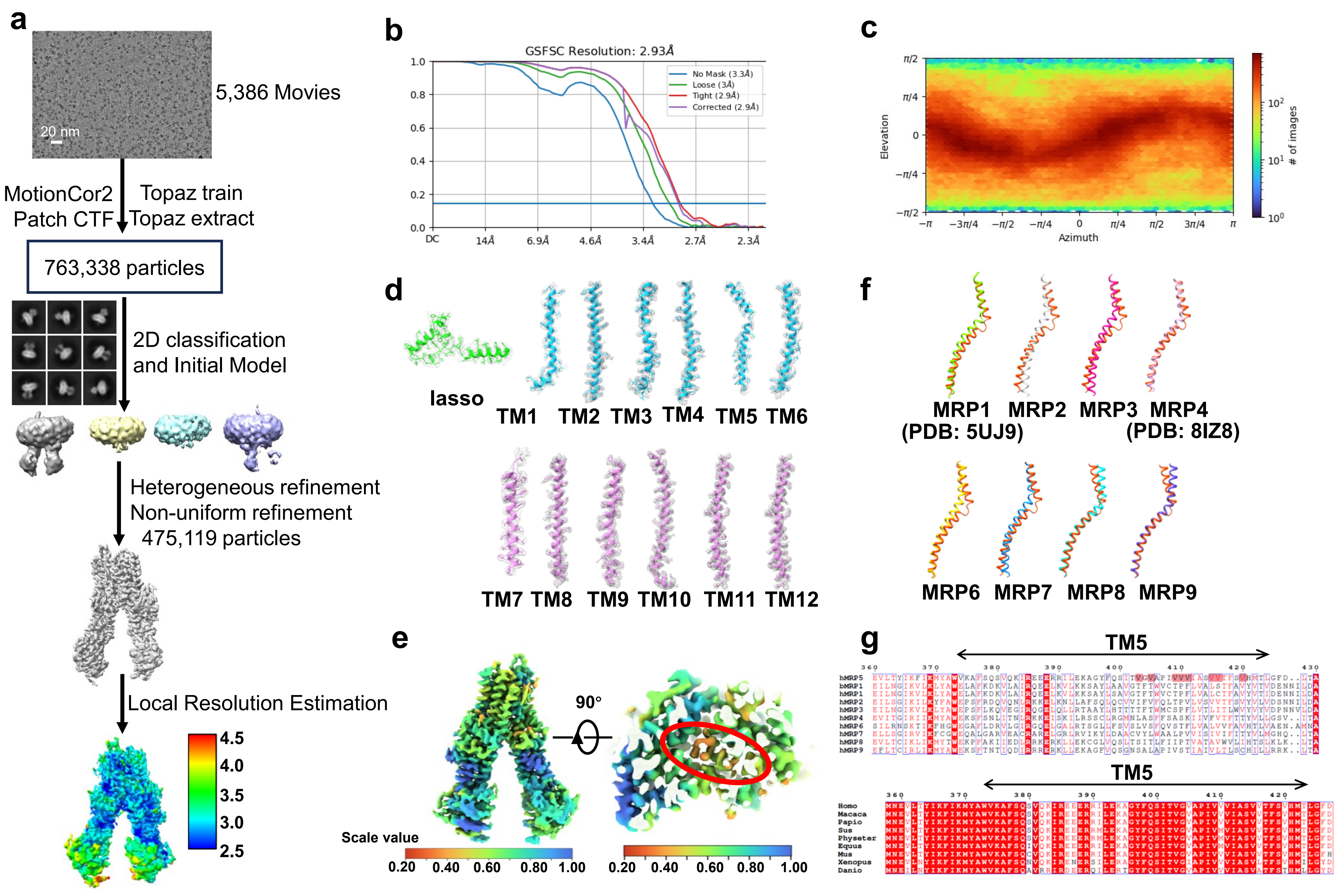
a
5,386 Movies
20 nm
MotionCor2
Patch CTF
Topaz train
Topaz extract
763,338 particles
2D classification
and Initial Model
Heterogeneous refinement
Non-uniform refinement
475,119 particles
Local Resolution Estimation
4.5
4.0
3.5
3.0
2.5
b
GSFSC Resolution: 2.93Å
No Mask (3.3Å)
Loose (3Å)
Tight (2.9Å)
Corrected (2.9Å)
c
Elevation
Azimuth
# of images
d
lasso
TM1 TM2 TM3 TM4 TM5 TM6
TM7 TM8 TM9 TM10 TM11 TM12
e
90°
Scale value
0.20 0.40 0.60 0.80 1.00
f
MRP1 MRP2 MRP3 MRP4
(PDB: 5UJ9) (PDB: 8IZ8)
MRP6 MRP7 MRP8 MRP9
g
TM5
hMRP5
bMRP1
hMRP1
hMRP2
hMRP3
hMRP4
hMRP6
hMRP7
hMRP8
hMRP9
TM5
Homo
Macaca
Papio
Sus
Physeter
Equus
Mus
Xenopus
Danio

Supplementary Fig.1 Data processing, model building and analysis for wt-hMRP5.

a) Flowchart for cryogenic electron microscopy data processing. Representative cryogenic electron microscopy micrographs and two-dimensional (2D) averages. Bar: 20 nm. The micrograph is representative of 5,386 cryogenic electron microscopy images.

b) Gold-standard Fourier shell correlation (GSFSC) curve for the wt-hMRP5 map generated using cryoSPARC 3.1.

c) Euler angle distribution of the classified particles used for the final three-dimensional refinement of the overall map.

d) Electron microscopy density of lasso domain and each transmembrane helix (TM) of wt-hMRP5. Contour levels is 0.217 (lasso), 0.244 (TM1), 0.217 (TM2), 0.217 (TM3), 0.224 (TM4), 0.231 (TM5), 0.231 (TM6), 0.217 (TM7), 0.224 (TM8), 0.224 (TM9), 0.224 (TM10), 0.217 (TM11) and 0.204 (TM12).

e) wt-hMRP5 map, annotated by the occupancy, and colored according to the estimated local scale.

f) Comparison of TM5 among hMRP5 (Indian red) and other MRP family members. The structures of bovine MRP1 (PDB ID: 5UJ9), human MRP4 (PDB: 8IZ8) and the predicted structures of human MRP2, MRP3, MRP6, MRP7, MRP8, and MRP9 (downloaded from alphafold.ebi.ac.uk) were used in the comparison analysis. The following proteins are colored: bovine MRP1 (Chartreuse), MRP2 (White), MRP3 (Deep pink), MRP4 (Plum), MRP6 (Gold), MRP7 (Dodger blue), MRP8 (Cyan), and MRP9 (Slate blue).

g) Sequences alignment of TM5 of human MRP subfamilies and MRP5 orthologs. Invariant and highly conserved residues are shaded red and colored red, respectively. The valines are shaded in Indian red.

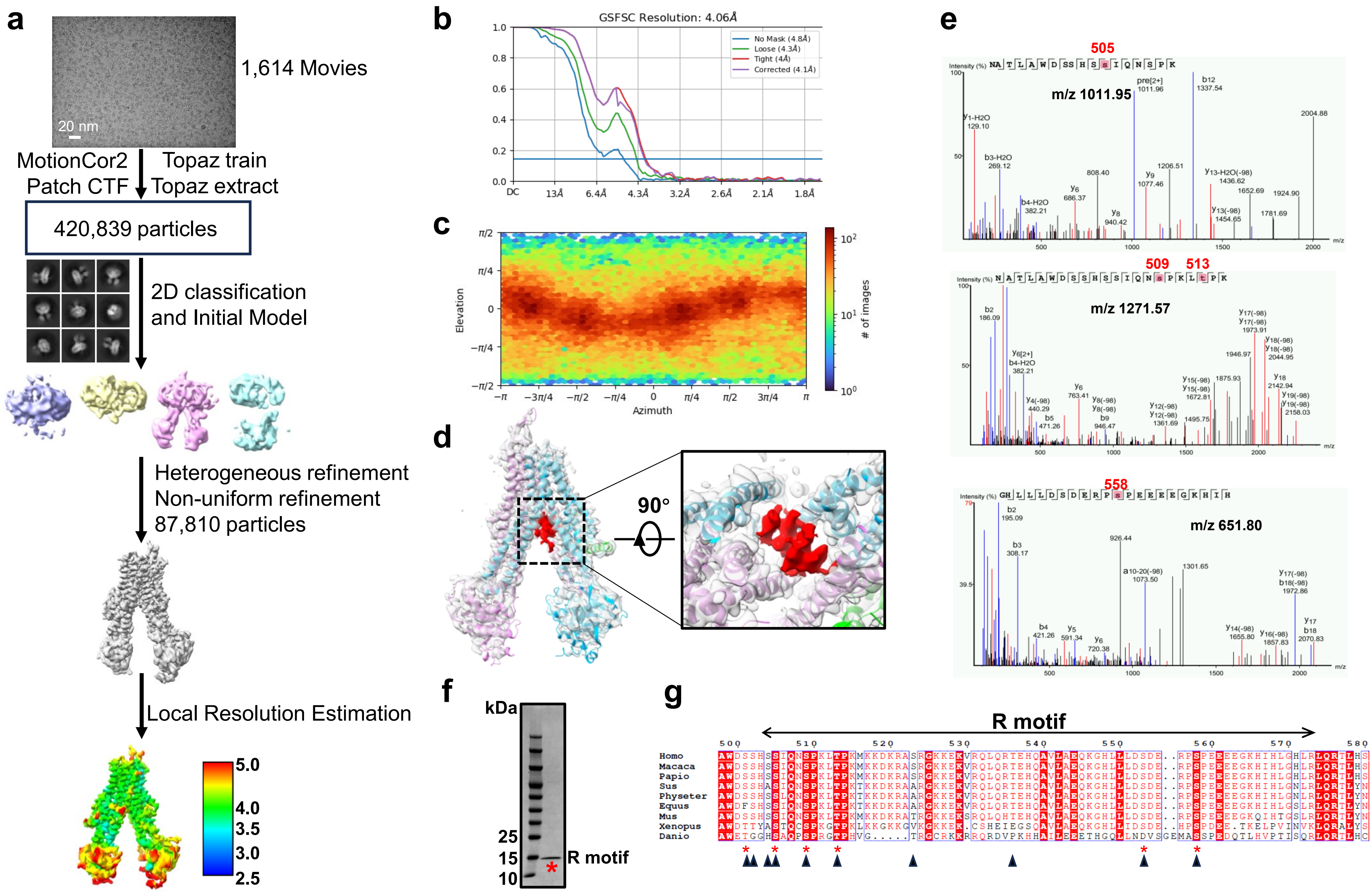
a
1,614 Movies
20 nm
MotionCor2
Patch CTF
Topaz train
Topaz extract
420,839 particles
2D classification and Initial Model
Heterogeneous refinement
Non-uniform refinement
87,810 particles
Local Resolution Estimation
5.0
4.0
3.5
3.0
2.5
b
GSFSC Resolution: 4.06Å
No Mask (4.8Å)
Loose (4.3Å)
Tight (4Å)
Corrected (4.1Å)
c
Elevation
Azimuth
# of images
d
90°
e
505
m/z 1011.95
509
513
m/z 1271.57
558
m/z 651.80
f
kDa
25
15
10
R motif
g
R motif
Homo
Macaca
Papio
Sus
Physeter
Equus
Mus
Xenopus
Danio

Supplementary Fig.2 Data processing, model building and analysis for hMRP5-ΔR.

a) Flowchart for cryogenic electron microscopy data processing. Representative cryogenic electron microscopy micrographs and two-dimensional (2D) averages. Bar: 20 nm. The micrograph is representative of 1,614 cryogenic electron microscopy images.

b) Gold-standard Fourier shell correlation (GSFSC) curve for the hMRP5-ΔR map generated using cryoSPARC 3.1.

c) Euler angle distribution of the classified particles used for the final three-dimensional refinement of the overall map.

d) Overlap of Electron microscopy density and cartoon of hMRP5-ΔR. Contour levels is 0.234.

e) Phosphorylation mass spectrometry analysis (biological replicate n=1) of wt-hMRP5, and selected phosphorylation sites, including Ser505, Ser509, Thr513, and Ser558.

f) SDS PAGE of R motif.

g) Multiple-sequence alignment for R motif of hMRP5 and orthologs. Invariant and highly conserved residues are shaded red and colored red, respectively. The predicted phosphorylation sites (https://d2p2.pro/search) of the R motif are marked with red asterisks and the O-glycosylation sites (https://services.healthtech.dtu.dk/service.php?YinOYang-1.2) are marked with black triangles at the bottom of sequences.

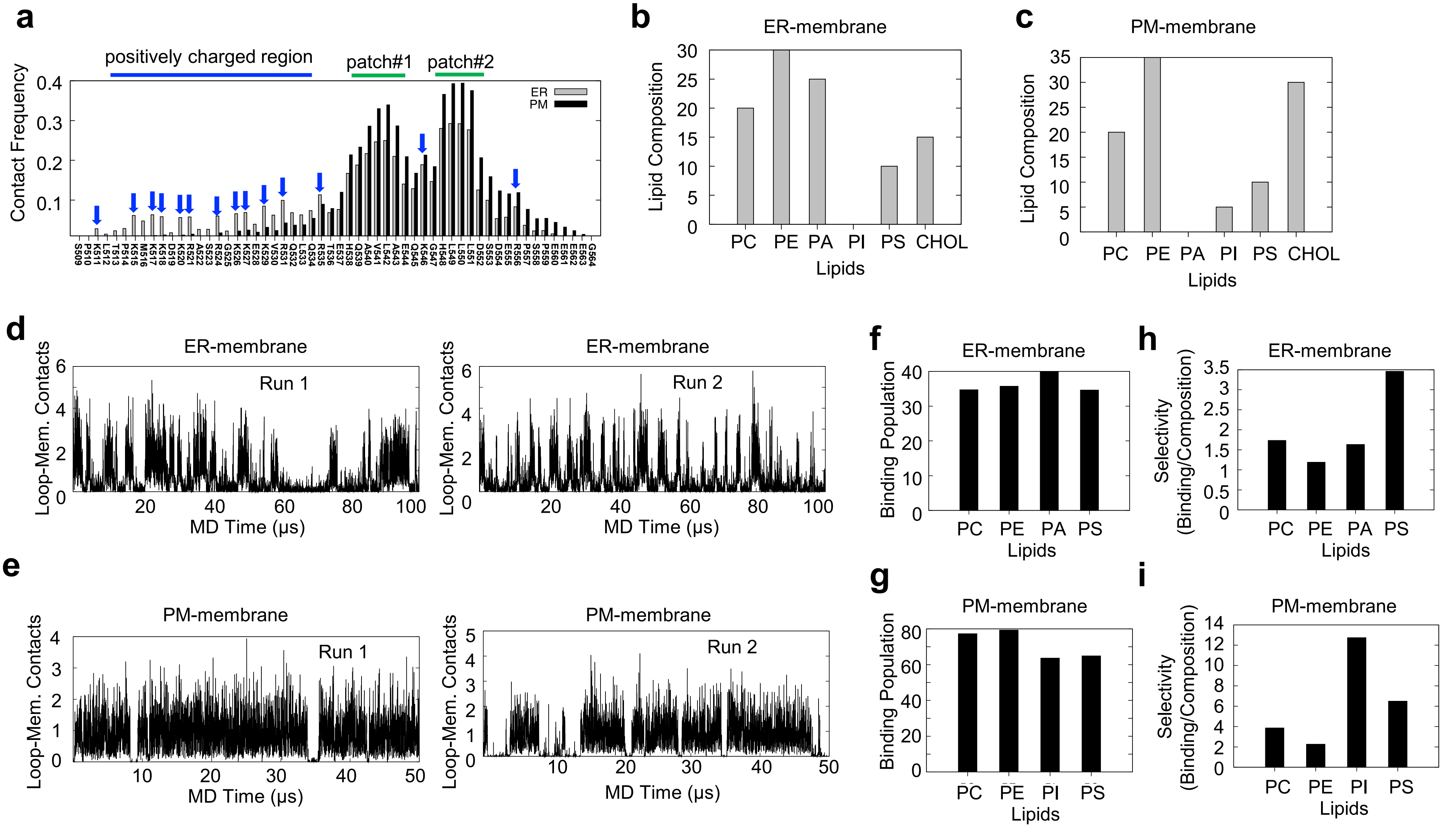
a
positively charged region
patch#1
patch#2
Contact Frequency
ER
PM
b
ER-membrane
Lipid Composition
PC PE PA PI PS CHOL
Lipids
c
PM-membrane
Lipid Composition
PC PE PA PI PS CHOL
Lipids
d
ER-membrane
Run 1
Run 2
Loop-Mem. Contacts
MD Time (μs)
e
PM-membrane
Run 1
Run 2
Loop-Mem. Contacts
MD Time (μs)
f
ER-membrane
Binding Population
PC PE PA PS
Lipids
g
PM-membrane
Binding Population
PC PE PI PS
Lipids
h
ER-membrane
Selectivity (Binding/Composition)
PC PE PA PS
Lipids
i
PM-membrane
Selectivity (Binding/Composition)
PC PE PI PS
Lipids

Supplementary Fig.3 Molecular dynamic simulation of R motif.

a) Contact frequency of the R-domain interacting with membranes resembling the ER and PM. Arrows in blue point out the regions rich in positively charged residues. The area with positive charge and the pair of hydrophobic areas are marked with lines in blue and green, respectively.

b) Composition of lipids in the simulated ER-like membrane.

c) Composition of lipids in the simulated PM-like membrane.

d) Trajectories of two coarse-grained molecular dynamics simulations (identified as RUN1 and RUN2) within an ER-like membrane, as a function of the contacts between the R-motif loop and the membrane. .

e) Trajectories of two coarse-grained molecular dynamics simulations (identified as RUN1 and RUN2) within a PM-like membrane, as a function of the contacts between the R-motif loop and the membrane. .

f) The observed binding frequency of the R motif with different lipids in the ER-like membrane.

g) The observed binding frequency of the R motif with different lipids in the PM-like membrane.

h) The lipid selectivity of the R motif with an ER-like membrane.

i) The lipid selectivity of the R motif with a PM-like membrane.

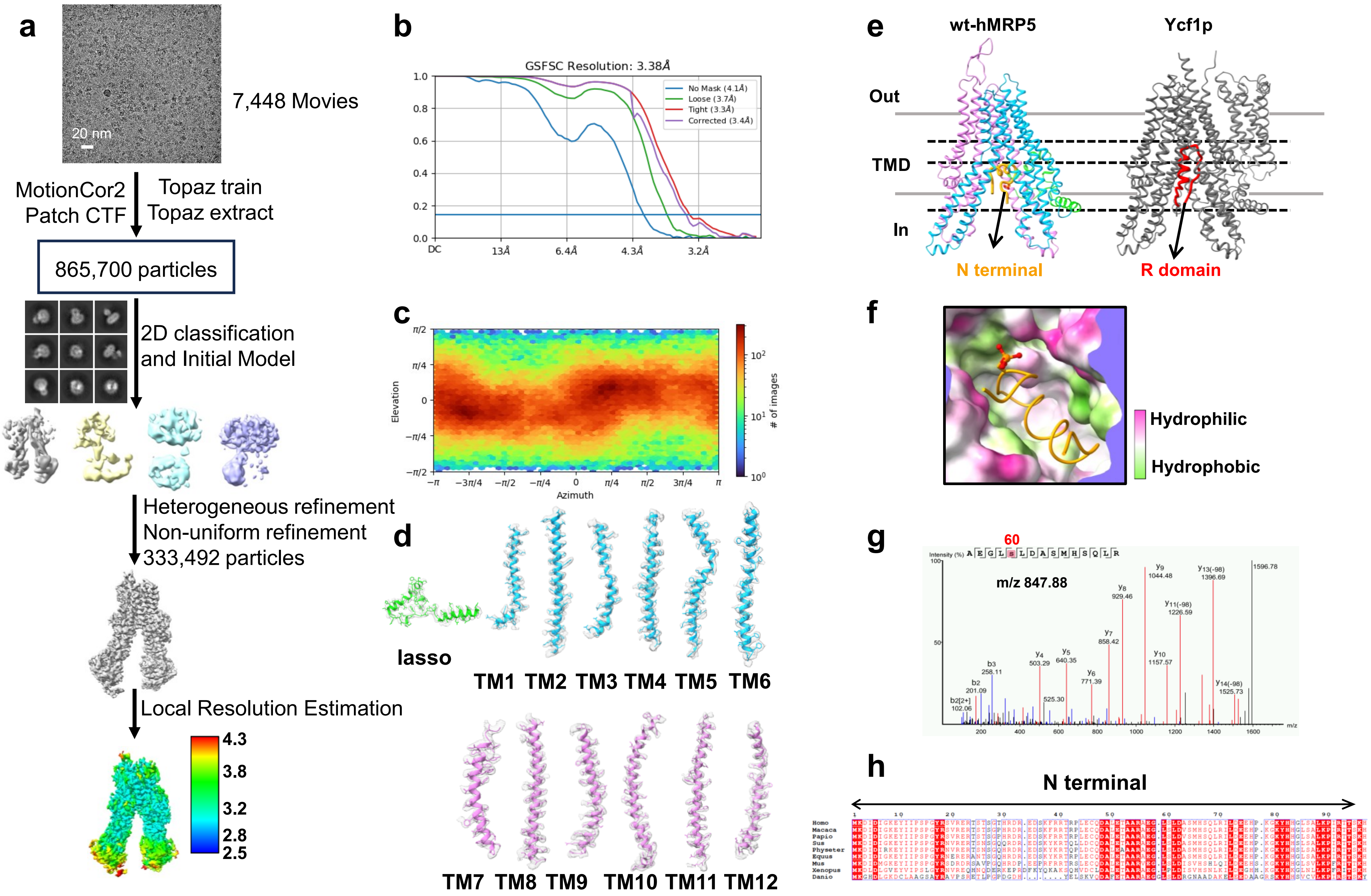
a
7,448 Movies
20 nm
MotionCor2
Patch CTF
Topaz train
Topaz extract
865,700 particles
2D classification
and Initial Model
Heterogeneous refinement
Non-uniform refinement
333,492 particles
Local Resolution Estimation
4.3
3.8
3.2
2.8
2.5
b
GSFSC Resolution: 3.38Å
No Mask (4.1Å)
Loose (3.7Å)
Tight (3.3Å)
Corrected (3.4Å)
DC
13Å
6.4Å
4.3Å
3.2Å
c
Elevation
Azimuth
# of images
d
lasso
TM1 TM2 TM3 TM4 TM5 TM6
TM7 TM8 TM9 TM10 TM11 TM12
e
wt-hMRP5
Ycf1p
Out
TMD
In
N terminal
R domain
f
Hydrophilic
Hydrophobic
g
60
m/z 847.88
h
N terminal
Homo
Macaca
Papio
Sus
Physeter
Equus
Mus
Xenopus
Danio

Supplementary Fig. 4 Data processing and model building for hMRP5-Δ1-94 and analysis for autoinhibited state.

a) Flowchart for cryogenic electron microscopy data processing. Representative cryogenic electron microscopy micrographs and two-dimensional (2D) averages. Bar: 20 nm. The micrograph is representative of 7,448 cryogenic electron microscopy images.

b) Gold-standard Fourier shell correlation (GSFSC) curve for the hMRP5-Δ1-94 map generated using cryoSPARC 3.1.

c) Euler angle distribution of the classified particles used for the final three-dimensional refinement of the overall map.

d) Electron microscopy density of lasso domain and each transmembrane helix (TM) of hMRP5-Δ1-94. Contour levels are 0.524 (lasso), 0.569 (TM1), 0.537 (TM2), 0.477 (TM3), 0.47 (TM4), 0.47 (TM5), 0.574 (TM6), 0.58 (TM7), 0.517 (TM8), 0.525 (TM9), 0.557 (TM10), 0.642 (TM11) and 0.568 (TM12).

e) The model comparison between wt-hMRP5 and dephosphorylated Ycf1p (PDB: 8SG4). The 46-64 peptide from N terminal of hMRP5 colored orange and the regulatory domain (R domain) from Ycf1p colored red.

f) The hydrophobic pocket of wt-hMRP5.

g) Phosphorylation mass spectrometry analysis of Ser60 of wt-hMRP5.

h) Sequences alignment of N terminal of MRP5 orthologs.

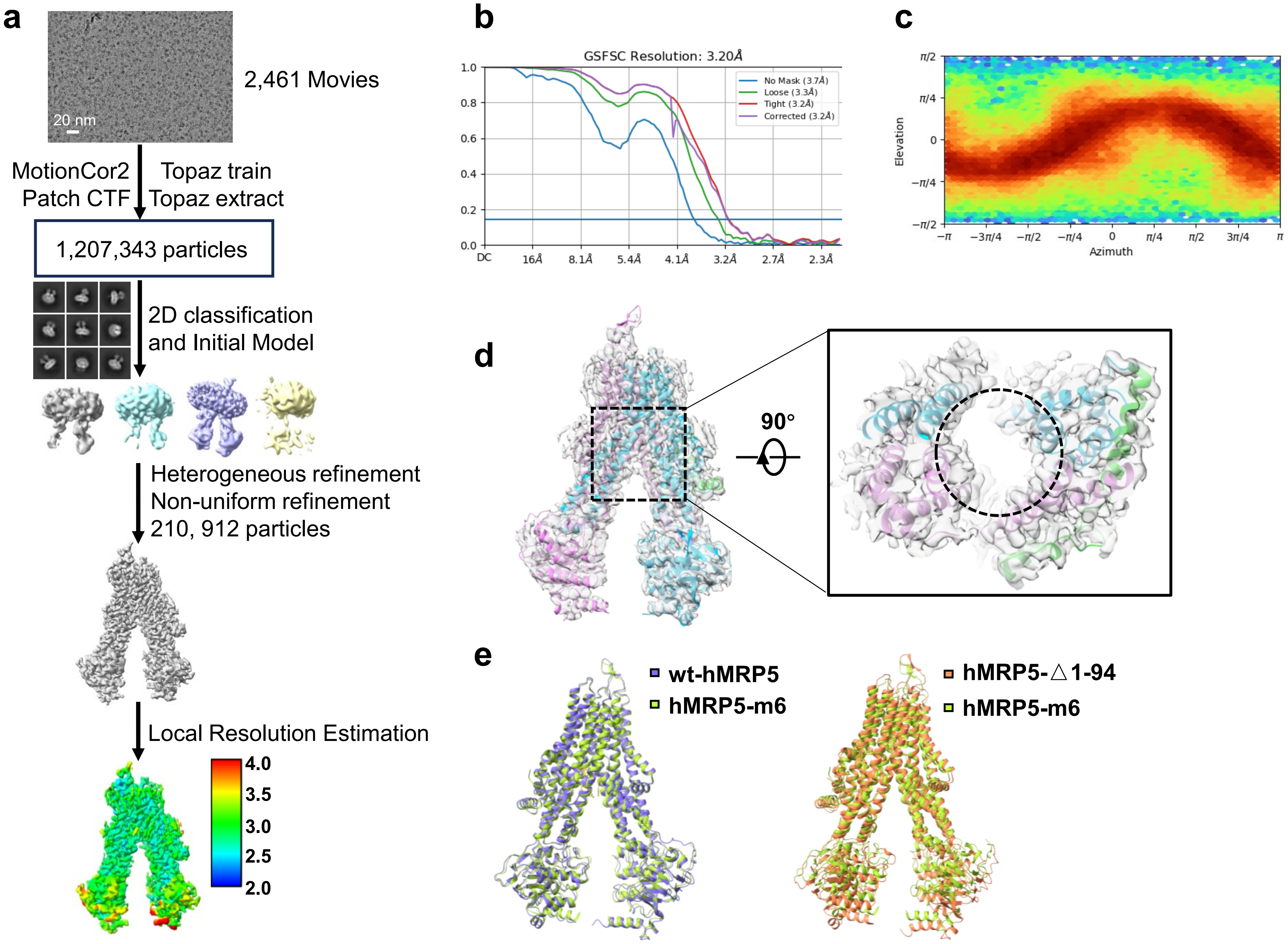
a
20 nm
2,461 Movies
MotionCor2
Patch CTF
Topaz train
Topaz extract
1,207,343 particles
2D classification
and Initial Model
Heterogeneous refinement
Non-uniform refinement
210, 912 particles
Local Resolution Estimation
4.0
3.5
3.0
2.5
2.0
b
GSFSC Resolution: 3.20Å
No Mask (3.7Å)
Loose (3.3Å)
Tight (3.2Å)
Corrected (3.2Å)
DC
16Å
8.1Å
5.4Å
4.1Å
3.2Å
2.7Å
2.3Å
c
Elevation
Azimuth
# of images
d
90°
e
wt-hMRP5
hMRP5-m6
hMRP5-△1-94
hMRP5-m6

Supplementary Fig. 5 Data processing, model building and analysis for hMRP5-m6.

a) Flowchart for cryogenic electron microscopy data processing. Representative cryogenic electron microscopy micrographs and two-dimensional (2D) averages. Bar: 20 nm. The micrograph is representative of 2,461 cryogenic electron microscopy images.

b) Gold-standard Fourier shell correlation (GSFSC) curve for the hMRP5-m6 map generated using cryoSPARC 3.1.

c) Euler angle distribution of the classified particles used for the final three-dimensional refinement of the overall map.

d) Electron microscopy density and cartoon of hMRP5-m6. Contour level is 0.427.

e) Superposition of wt-hMRP5, hMRP5-m6, and hMRP5-Δ1-94.

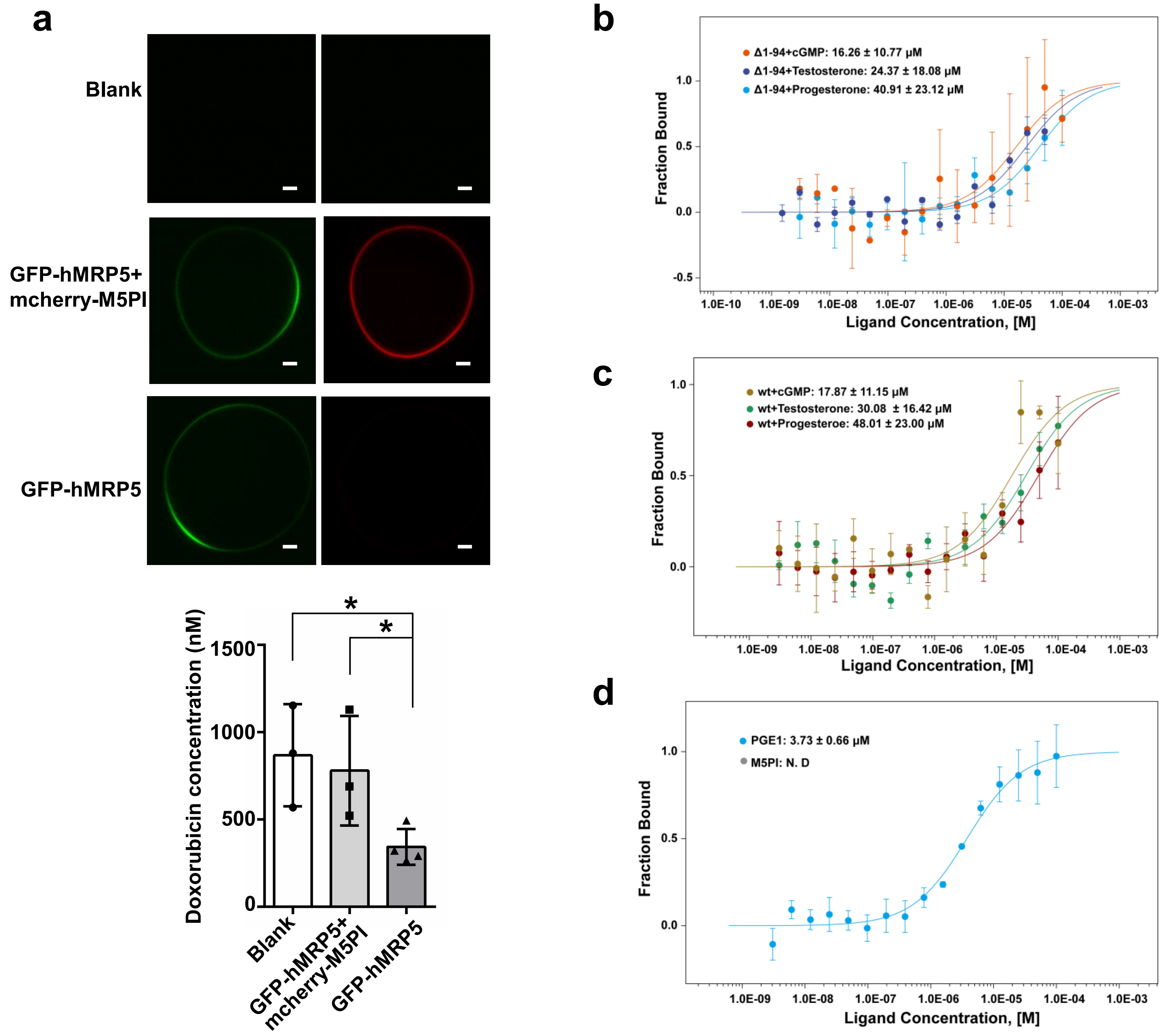
a
Blank
GFP-hMRP5+
mcherry-M5PI
GFP-hMRP5
Doxorubicin concentration (nM)
1500
1000
500
0
*
*
Blank
GFP-hMRP5+
mcherry-M5PI
GFP-hMRP5
b
Δ1-94+cGMP: 16.26 ± 10.77 μM
Δ1-94+Testosterone: 24.37 ± 18.08 μM
Δ1-94+Progesterone: 40.91 ± 23.12 μM
Fraction Bound
1.0
0.5
0.0
-0.5
1.0E-10
1.0E-09
1.0E-08
1.0E-07
1.0E-06
1.0E-05
1.0E-04
1.0E-03
Ligand Concentration, [M]
c
wt+cGMP: 17.87 ± 11.15 μM
wt+Testosterone: 30.08 ± 16.42 μM
wt+Progesteroe: 48.01 ± 23.00 μM
Fraction Bound
1.0
0.5
0.0
1.0E-09
1.0E-08
1.0E-07
1.0E-06
1.0E-05
1.0E-04
1.0E-03
Ligand Concentration, [M]
d
PGE1: 3.73 ± 0.66 μM
M5PI: N. D
Fraction Bound
1.0
0.5
0.0
1.0E-09
1.0E-08
1.0E-07
1.0E-06
1.0E-05
1.0E-04
1.0E-03
Ligand Concentration, [M]

Supplementary Fig. 6 Transport activity of hMRP5 and MST assay of hMRP5 and hMRP4.

a) GFP fluorescence indicates the expression of genes encoding GFP tagged hMRP5 and mcherry fluorescence indicates the expression of genes encoding mcherry tagged M5PI. Water was used as a blank control. Independent experiments have been repeated three times with similar results. Bar=100 μm. The y axis is the doxorubicin concentration in the final 50 μl extract of Xenopus oocytes for mass spectrometry analysis. Bars are mean ± SD. Points represent biologically independent experiments (n=3 for blank control and GFP-hMRP5+mcherry-M5PI group, n=4 for GFP-hMRP5 group). Two-tailed t-test was performed. **P* = 0.0190 between blank and GFP-hMRP5; **P* = 0.0442 between GFP-hMRP5+mcherry-M5PI and GFP-hMRP5.

b) The binding affinity between hMRP5-Δ1-94 and substrates. Data represent mean ± SEM of three independent measurements.

c) The binding affinity between wt-hMRP5 and substrates. Data represent mean ± SEM of three independent measurements.

d) The binding affinity between hMRP4 and M5PI or PGE1. Data represent mean ± SEM of three independent measurements. N. D: not detectable.

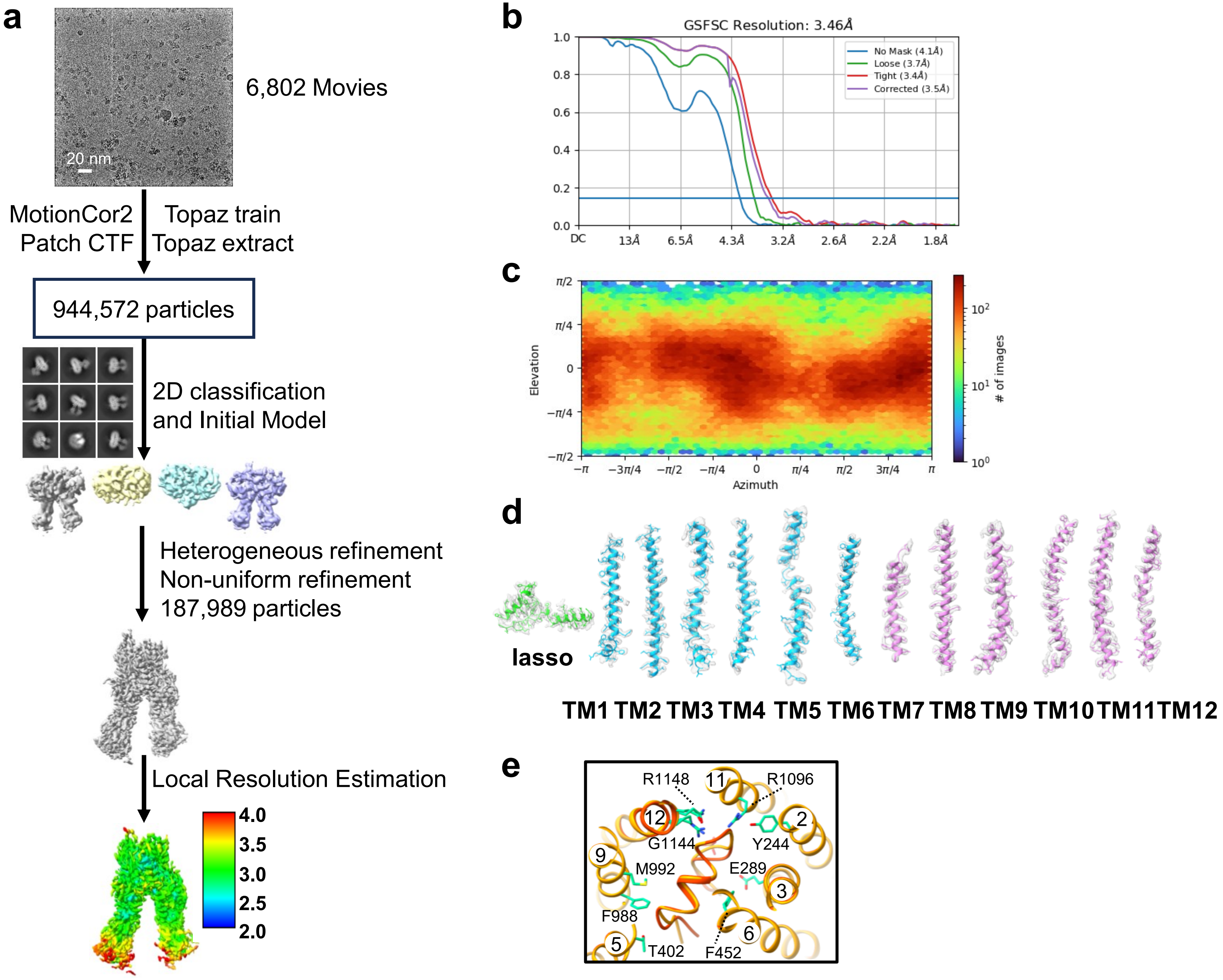

a
20 nm
6,802 Movies
MotionCor2
Patch CTF
Topaz train
Topaz extract
944,572 particles
2D classification
and Initial Model
Heterogeneous refinement
Non-uniform refinement
187,989 particles
Local Resolution Estimation
4.0
3.5
3.0
2.5
2.0
b
GSFSC Resolution: 3.46Å
No Mask (4.1Å)
Loose (3.7Å)
Tight (3.4Å)
Corrected (3.5Å)
DC
13Å
6.5Å
4.3Å
3.2Å
2.6Å
2.2Å
1.8Å
c
Elevation
Azimuth
# of images
d
lasso
TM1 TM2 TM3 TM4 TM5 TM6 TM7 TM8 TM9 TM10 TM11 TM12
e
R1148
R1096
G1144
Y244
M992
E289
F988
T402
F452

Supplementary Fig. 7 Data processing and model building for M5PI-bound hMRP5.

a) Flowchart for cryogenic electron microscopy data processing. Representative cryogenic electron microscopy micrographs and two-dimensional (2D) averages. Bar: 20 nm. The micrograph is representative of 6,802 cryogenic electron microscopy images.

b) Gold-standard Fourier shell correlation (GSFSC) curve for the M5PI-bound hMRP5 map generated using cryoSPARC 3.1.

c) Euler angle distribution of the classified particles used for the final three-dimensional refinement of the overall map.

d) Electron microscopy density of lasso domain and each transmembrane helix (TM) of M5PI-bound hMRP5. Contour levels are 0.29 (lasso), 0.62 (TM1), 0.649 (TM2), 0.472 (TM3), 0.406 (TM4), 0.461 (TM5), 0.511 (TM6), 0.472 (TM7), 0.457 (TM8), 0.395 (TM9), 0.44 (TM10), 0.642 (TM11) and 0.568 (TM12).

e) Superposition of wt-hMRP5 (orange) and M5PI-bound hMRP5 (orange red). The common contact sites between these two structures are shown as sticks and colored in medium spring green by heteroatoms.

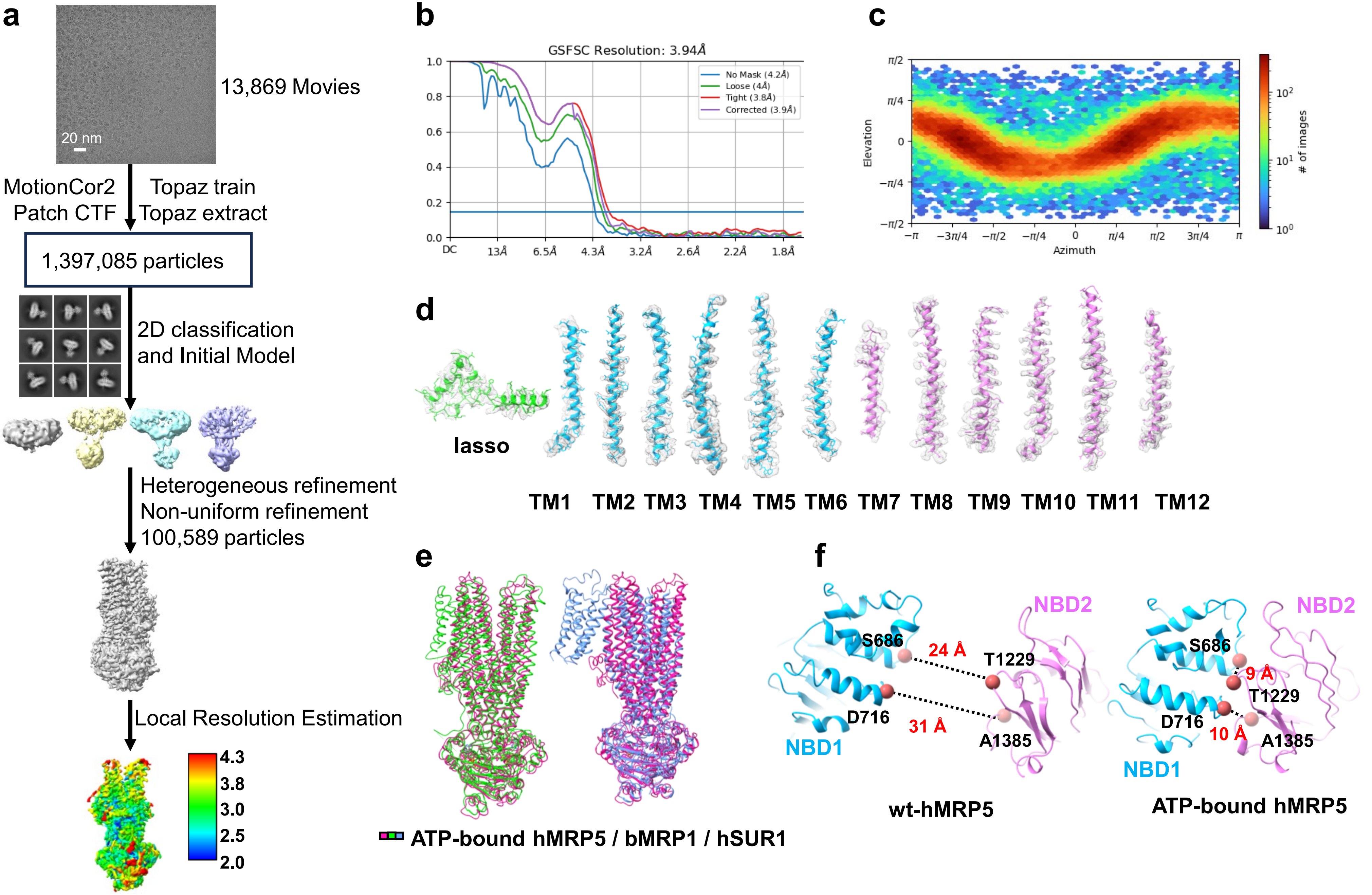
a
13,869 Movies
20 nm
MotionCor2
Patch CTF
Topaz train
Topaz extract
1,397,085 particles
2D classification
and Initial Model
Heterogeneous refinement
Non-uniform refinement
100,589 particles
Local Resolution Estimation
4.3
3.8
3.0
2.5
2.0
b
GSFSC Resolution: 3.94Å
No Mask (4.2Å)
Loose (4Å)
Tight (3.8Å)
Corrected (3.9Å)
c
Elevation
Azimuth
# of images
d
lasso
TM1 TM2 TM3 TM4 TM5 TM6 TM7 TM8 TM9 TM10 TM11 TM12
e
ATP-bound hMRP5 / bMRP1 / hSUR1
f
NBD1
NBD2
S686
T1229
D716
A1385
24 Å
31 Å
9 Å
10 Å
wt-hMRP5
ATP-bound hMRP5

Supplementary Fig. 8 Data processing, model building and analysis for ATP-bound hMRP5.

a) Flowchart for cryogenic electron microscopy data processing. Representative cryogenic electron microscopy micrographs and two-dimensional (2D) averages. Bar: 20 nm. The micrograph is representative of 13,869 cryogenic electron microscopy images.

b) Gold-standard Fourier shell correlation (GSFSC) curve for the ATP-bound hMRP5 map generated using cryoSPARC 3.1.

c) Euler angle distribution of the classified particles used for the final three-dimensional refinement of the overall map.

d) Electron microscopy density of lasso domain and each transmembrane helix (TM) of ATP-bound hMRP5. Contour levels are 0.643 (lasso), 0.584 (TM1), 0.62 (TM2), 0.53 (TM3), 0.573 (TM4), 0.574 (TM5), 0.592 (TM6), 0.56 (TM7), 0.53 (TM8), 0.592 (TM9), 0.637 (TM10), 0.637 (TM11), and 0.574 (TM12).

e) Alignment of ATP-bound structure of hMRP5 (deep pink), bMRP1 (lime) (PDB: 6BHU), and hSUR1 (medium blue) (PDB: 6C3O), and ATP-bound structure of hMRP5 and bMRP1 show outward-open conformation.

f) The ATP-bound structure of hSUR1 shows outward-occluded conformation. The NBDs comparison between wt-hMRP5 (left) and ATP-bound hMRP5 (right). The distance between Ser686 of NBD1 and Thr1229 of NBD2, Asp716 of NBD1 and Ala1385 of NBD2 are indicated.

Supplementary Table 1 Molecular dynamics simulations checklist.

Reliability and reproducibility checklist for molecular dynamics simulations<br><br>***All boxes must be marked YES by acceptance unless an N/A option is available**	Yes	N/A	Response<br>(Please state where this information can be found in the text)
**1. Convergence of simulations and analysis**			
1a. Is an evaluation presented in the text to show that the property being measured has equilibrated in the simulations (*e.g.* time-course analysis)?	☒		Yes, the evaluation is detailed in Methods section, and is further illustrated in Figures 3c and Supplementary Figure 3.
1b. Then, is it described in the text how simulations are split into equilibration and production runs and how much data were analyzed from production runs?	☒		Yes, the division of simulations into equilibration and production phases, along with the extent of data analysis from the production runs, is outlined in the "Molecular Dynamics Simulation" subsection within the Methods section.
1c. Are there at least 3 simulations per simulation condition with statistical analysis?	☐		There are two 1000ns simulations for each condition in the AA MD and two 50 us or 100 us simulations in the CG MD, complete with statistical analysis, as detailed in Figures 3c and Supplementary Figure 3, and discussed in the "Molecular Dynamics Simulation" subsection of the Methods section.
1d. Is evidence provided in the text that the simulation results presented are independent of initial configuration?	☒		Figure 3c and Supplementary Figure 3.
**2. Connection to experiments**			
2a. Are calculations provided that can connect to experiments (*e.g.* loss or gain in function from mutagenesis, binding assays, NMR chemical shifts, J-couplings, SAXS curves, interaction distances or FRET distances, structure factors, diffusion coefficients, bulk modulus and other mechanical properties, *etc.*)?	☒		Yes, our study includes calculations that can be correlated with mutagenesis experimental.
**3. Method choice**			
3a. Is it described in the text what force field and water model are used and why?	☒		Yes, the suitability and accuracy of our chosen models for the questions under investigation are discussed, with a specific emphasis on the use of the CHARMM36m force field, as detailed in the "Molecular Dynamics Simulation" subsection of the Methods section.
3b. Do simulations contain membranes, membrane proteins, intrinsically disordered proteins, glycans, nucleic acids, polymers, or cryptic ligand binding?	☒	☐	Yes, our simulations specifically include both membranes and membrane proteins, focusing on these crucial components to accurately model and understand their dynamics and interactions within the cellular environment.
If 3b is **YES**, are enhanced sampling methods used?	☐	☒	Response not needed if **N/A**
If enhanced sampling methods are used, are the convergence criteria clearly stated?	☐		
If 3b is **YES**, is it explained in the text why or why not enhanced sampling methods are used?	☒		Yes, evidence supporting the adequacy of brute-force CG MD simulation timescales for our study is provided in detail in Methods section, and visually represented in Figure 3c and Supplementary Figure 3.
**4. Code and reproducibility**			
4a. Is a table provided describing the system setup, such as simulation box dimensions, total number of atoms, total number of water molecules, salt concentration, lipid composition (number of molecules and type)?	☐	No	This information is comprehensively detailed in the "Molecular Dynamics Simulation" subsection of the Methods section.
4b. Is it described in the text what simulation and analysis software and which versions are used?	☒		Yes. In Method section, we specify the simulation and analysis software employed, along with their respective versions.
4c. Are initial coordinate and simulation input files and a coordinate file of the final output provided as supplementary files or in a public repository?	☒		Yes. The files have been uploaded to GitHub and are accessible via the following link: https://github.com/yongwangCPH/papers/tree/main/2024/MRP5
4d. Is there custom code or custom force field parameters?	☐	☒	Response not needed if **N/A**
If **YES**, are they provided as supplementary profiles or in a public repository?	☐		

Supplementary Table 2 Overview of cryo-EM data collection and coordinate refinement.

	wt-hMRP5	hMRP5-ΔR	hMRP5-Δ1-94	hMRP5-m6	M5PI-bound hMRP5	ATP-bound hMRP5
**Data collection and processing**						
Magnification	81,000×	105,000×	105,000×	81,000×	96,000×	96,000×
Voltage (kV)	300	300	300	300	300	300
Camera	Gatan K3 Summit	Gatan K3 Summit	Gatan K3 Summit	Gatan K3 Summit	Falcon4	Falcon4
Camera mode	Super-resolution	Super-resolution	Super-resolution	Super-resolution	Super-resolution	Super-resolution
Electron exposure ($e^-/Å^2$)	50	50	50	50	50	50
Defocus range (μm)	-1.2 to -0.8	-1.2 to -0.8	-1.2 to -0.8	-1.2 to -0.8	-1.2 to -0.8	-1.2 to -0.8
Pixel size (Å)	1.072	0.855	1.29	1.08	0.82	0.86
Movies	5,386	1,614	7,448	2,461	6,802	13,869
Frames/movie	32	32	32	32	32	32
Symmetry imposed	C1	C1	C1	C1	C1	C1
Initial particle projections (no.)	763,338	420,839	865,700	1,207,343	944,572	1,397,085
Final particle projections (no.)	475, 119	87,810	333,492	210, 912	187,989	100,589
Map resolution (Å)	2.93	4.06	3.38	3.20	3.46	3.94
FSC threshold	0.143	0.143	0.143	0.143	0.143	0.143
Map resolution range (Å)	2.5 to 4.5	2.5 to 5.0	2.5 to 4.3	2.0 to 4.0	2.0 to 4.0	2.0 to 4.3
Initial model used	Not applicable (N/A)	Not applicable (N/A)	Not applicable (N/A)	Not applicable (N/A)	Not applicable (N/A)	Not applicable (N/A)
**Refinement**						
Model resolution (Å)	2.93	4.06	3.38	3.2	3.46	3.94
FSC threshold	0.143	0.143	0.143	0.143	0.143	0.143
Map sharpening B factor ($Å^2$)	-137.1	-156.5	-148.4	-140.5	-158.3	-159.2
**Model composition**						
Non-hydrogen atoms	9717	9571	9489	9443	9735	9515
Protein residues	1240	1219	1208	1207	1242	1240
Ligand	/	/	/	/	M5PI	ATP, Mg^{2+}
**R.m.s. deviations**						
Bond lengths (Å)	0.26	0.25	0.41	0.24	0.24	0.43
Bond angles (°)	0.48	0.52	0.60	0.48	0.46	0.61
MolProbity score	1.40	1.78	1.96	1.92	1.44	1.89
Clashscore	9	11	6	8	6	9
Rotamer outliers (%)	0.2	0.0	0.1	0.5	0.0	0.3
**Ramachandran plot**						
Favored (%)	97	97	93	94	98	90
Allowed (%)	3	3	7	5	2	10
Disallowed (%)	0	0	0	1	0	0

Source Data for Supplementary Fig. 2f

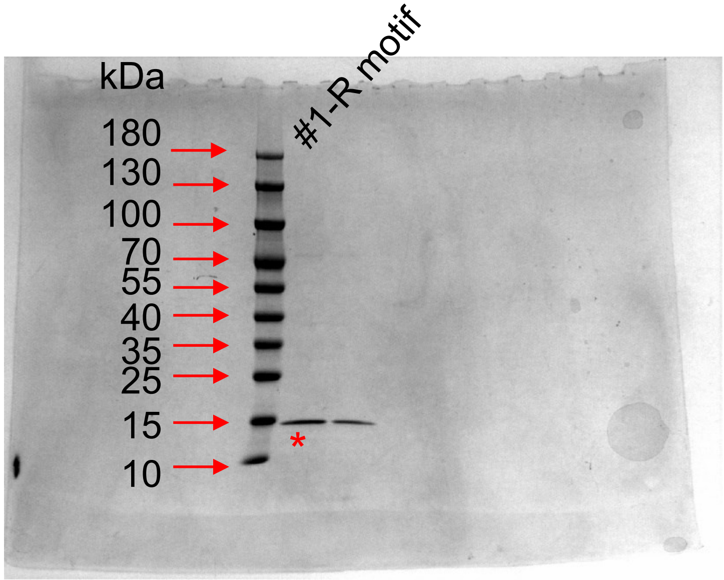

Supplement: Supplementary file 1 — Supplementary Information [file 41467_2024_49204_MOESM1_ESM.pdf]
