# Supplementary material for: Inhibition and transport mechanisms of the ABC transporter hMRP5
Source: Nat Commun. 2024 Jun 6;15:4811. doi: 10.1038/s41467-024-49204-1 (PMC11156954; doi:10.1038/s41467-024-49204-1)
Supplement: Supplementary file 3 — Description of Additional Supplementary Files [file 41467_2024_49204_MOESM3_ESM.pdf]

Description of Additional Supplementary Files

File name: Supplementary Data 1-4
Description: Supplementary Data 1-List of plasmids used in the study; Supplementary Data 2-List of primers used in the study; Supplementary Data 3- Mass spectrometry data of transport assay; Supplementary Data 4- Phosphorylation mass spectrometry data.

File name: Supplementary Movie 1
Description: R motif movement.
Coarse-grained molecular dynamics simulation of wt-hMRP5 in an ER-like membrane. The R motif of hMRP5 is shown as a green tube, Arginine and Lysine residues are depicted as blue sticks, and membrane lipids are represented by sticks. Additionally, lipid phosphate headgroups are highlighted as spheres, with phosphatidic acid PA) lipid headgroups in magenta and phosphatidylserine (PS) lipid headgroups in red.

File name: Supplementary Movie 2
Description: C46-S64 movement.
An all-atom molecular dynamics simulation of hMRP5 bound to the C46-S64 peptide. The membrane bilayer is depicted through the phosphorus atoms of the lipids. The hMRP5 protein itself is represented as a grey, semi-transparent cartoon. Highlighted within this structure, the R motif is colored green for emphasis. The C46-S64 peptide is illustrated using spheres, which are color-graded from blue at the N-terminal to red at the C-terminal, delineating its orientation. Surrounding residues of hMRP5 are shown in line representation.
