# Supplementary material for: Inhibition and transport mechanisms of the ABC transporter hMRP5
Source: Nat Commun. 2024 Jun 6;15:4811. doi: 10.1038/s41467-024-49204-1 (PMC11156954; doi:10.1038/s41467-024-49204-1)
Supplement: Supplementary file 7 — Reporting Summary [file 41467_2024_49204_MOESM7_ESM.pdf]

nature portfolio

Corresponding author(s): Zhongmin Liu

Last updated by author(s): May 7, 2024

Reporting Summary

Nature Portfolio wishes to improve the reproducibility of the work that we publish. This form provides structure for consistency and transparency in reporting. For further information on Nature Portfolio policies, see our Editorial Policies and the Editorial Policy Checklist.

Statistics

For all statistical analyses, confirm that the following items are present in the figure legend, table legend, main text, or Methods section.

n/a	Confirmed	
☐	☒	The exact sample size (*n*) for each experimental group/condition, given as a discrete number and unit of measurement
☐	☒	A statement on whether measurements were taken from distinct samples or whether the same sample was measured repeatedly
☐	☒	The statistical test(s) used AND whether they are one- or two-sided<br>*Only common tests should be described solely by name; describe more complex techniques in the Methods section.*
☒	☐	A description of all covariates tested
☒	☐	A description of any assumptions or corrections, such as tests of normality and adjustment for multiple comparisons
☐	☒	A full description of the statistical parameters including central tendency (e.g. means) or other basic estimates (e.g. regression coefficient) AND variation (e.g. standard deviation) or associated estimates of uncertainty (e.g. confidence intervals)
☐	☒	For null hypothesis testing, the test statistic (e.g. *F*, *t*, *r*) with confidence intervals, effect sizes, degrees of freedom and *P* value noted<br>*Give P values as exact values whenever suitable.*
☒	☐	For Bayesian analysis, information on the choice of priors and Markov chain Monte Carlo settings
☒	☐	For hierarchical and complex designs, identification of the appropriate level for tests and full reporting of outcomes
☐	☒	Estimates of effect sizes (e.g. Cohen's *d*, Pearson's *r*), indicating how they were calculated

Our web collection on statistics for biologists contains articles on many of the points above.

Software and code

Policy information about availability of computer code

Data collection	cryo-EM data: EPU 2.8.1;<br>Western blot : Tanon 6100;<br>Microscopy: Zeiss Imager ZEN 3.8; Nikon Elements 5.3.
Data analysis	MotionCor2 v1.4.2; cryoSPARC v3.2; Coot v0.8.9; Phenix v1.13; Pymol v2.2; ChimeraX v1.4; Martini v2.2; Force filed; Gromacs v2020.3 or v2020.0; Graphpad Prism v7.0; Xcaliburv v4.0; MO.Affinity Analysis (x86); ImageJ v1.6.0.

For manuscripts utilizing custom algorithms or software that are central to the research but not yet described in published literature, software must be made available to editors and reviewers. We strongly encourage code deposition in a community repository (e.g. GitHub). See the Nature Portfolio guidelines for submitting code & software for further information.

Data

Policy information about availability of data

All manuscripts must include a data availability statement. This statement should provide the following information, where applicable:

- Accession codes, unique identifiers, or web links for publicly available datasets
- A description of any restrictions on data availability
- For clinical datasets or third party data, please ensure that the statement adheres to our policy

Cryo-EM maps were deposited in the Electron Microscopy Data Bank under accession codes EMD-37554 [https://www.ebi.ac.uk/emdb/EMD-37554] (wt-hMRP5),

EMD-37556 [https://www.ebi.ac.uk/emdb/EMD-37556] (hMRP5-Δ1-94), EMD-37555 [https://www.ebi.ac.uk/emdb/EMD-37555] (hMRP5-ΔR), EMD-37557 [https://www.ebi.ac.uk/emdb/EMD-37557] (hMRP5-m6), EMD-37558 [https://www.ebi.ac.uk/emdb/EMD-37558] (M5PI-bound hMRP5), and EMD-37105 [https://www.ebi.ac.uk/emdb/EMD-37105] (ATP-bound hMRP5). Atomic coordinates have been deposited into the PDB under accession numbers 8WI0 [https://doi.org/10.2210/pdb8WI0/pdb] (wt-hMRP5), 8WI3 [https://doi.org/10.2210/pdb8WI3/pdb] (hMRP5-Δ1-94), 8WI2 [https://doi.org/10.2210/pdb8WI2/pdb] (hMRP5-ΔR), 8WI4 [https://doi.org/10.2210/pdb8WI4/pdb] (hMRP5-m6), 8WI5 [https://doi.org/10.2210/pdb8WI5/pdb], (M5PI-bound hMRP5), and 8KCI [https://doi.org/10.2210/pdb8KCI/pdb] (ATP-bound hMRP5). Source data are provided with this paper.

Research involving human participants, their data, or biological material

Policy information about studies with human participants or human data. See also policy information about sex, gender (identity/presentation), and sexual orientation and race, ethnicity and racism.

Reporting on sex and gender	none
Reporting on race, ethnicity, or other socially relevant groupings	none
Population characteristics	none
Recruitment	none
Ethics oversight	none

Note that full information on the approval of the study protocol must also be provided in the manuscript.

Field-specific reporting

Please select the one below that is the best fit for your research. If you are not sure, read the appropriate sections before making your selection.

☒ Life sciences ☐ Behavioural & social sciences ☐ Ecological, evolutionary & environmental sciences

For a reference copy of the document with all sections, see nature.com/documents/nr-reporting-summary-flat.pdf

Life sciences study design

All studies must disclose on these points even when the disclosure is negative.

Sample size	For each hMRP5 complex, one cryo-EM dataset comprising several thousand micrographs were collected (shown in Supplementary Table 1). The number of no specific statistical methods were used to determine sample size as it was not generally applicable to our study. For electron microscopy data, sample size was determined by the availability of areas to image on grids. Sample sizes for immunostaining assays and transport assay were selected so as to power non-parametric statistical analyses.
Data exclusions	No data were excluded.
Replication	Data were either pooled from at least three replicate experiments; or representative data are shown. The number of biological replicates were provided in the figure legends.
Randomization	Allocation of Xenopus laevis was randomized.
Blinding	No blinding was performed. The samples were prepared, treated and analysed by the same standard procedure. The investigators did not expect the experimental results and most of the test data was automatically generated by the instrument.

Reporting for specific materials, systems and methods

We require information from authors about some types of materials, experimental systems and methods used in many studies. Here, indicate whether each material, system or method listed is relevant to your study. If you are not sure if a list item applies to your research, read the appropriate section before selecting a response.

Materials & experimental systems

n/a	Involved in the study	
☐	☒	Antibodies
☐	☒	Eukaryotic cell lines
☒	☐	Palaeontology and archaeology
☐	☒	Animals and other organisms
☒	☐	Clinical data
☒	☐	Dual use research of concern
☒	☐	Plants

Methods

n/a	Involved in the study	
☒	☐	ChIP-seq
☒	☐	Flow cytometry
☒	☐	MRI-based neuroimaging

Antibodies

Antibodies used	Anti-strep antibody (Shenggong Biotech, D191106, 1:5000)
Validation	The anti-strep antibody used in western blot were validated by the manufacturer (https://store.sangon.com/productDetail?productInfo.code=D191106).

Eukaryotic cell lines

Policy information about cell lines and Sex and Gender in Research

Cell line source(s)	HEK293F cells and 293T cells were gifts from Prof. Hong-Wei Wang (Tsinghua University, Beijing, China).
Authentication	No
Mycoplasma contamination	The cell lines were not tested for Mycoplasma contamination.
Commonly misidentified lines (See ICLAC register)	No

Animals and other research organisms

Policy information about studies involving animals; ARRIVE guidelines recommended for reporting animal research, and Sex and Gender in Research

Laboratory animals	X. laevis frogs were purchased from Nasco and bred in an in-house facility. Xenopus laevis (two to three years old) females were used for oocytes isolation.
Wild animals	No wild animals were used in this study.
Reporting on sex	This study did not involve sex-based analysis.
Field-collected samples	This study did not involve sample collected from the field.
Ethics oversight	All animal procedures were conducted following intemmational standards and were approved by the Animal Care and Use Committeeofthe Southemn University of Science and Technology.

Note that full information on the approval of the study protocol must also be provided in the manuscript.
